# The association between proton pump inhibitor use and risk of post-hospitalization acute kidney injury: a multicenter prospective matched cohort study

**DOI:** 10.1186/s12882-023-03211-4

**Published:** 2023-05-26

**Authors:** Yue Zhang, Nasrollah Ghahramani, Hadie Razjouyan, Djibril M. Ba, Vernon M. Chinchilli

**Affiliations:** 1 Department of Public Health Sciences, Penn State College of Medicine, 90 Hope Drive, Hershey, PA 17033 USA; 2 Department of Medicine, Penn State College of Medicine, Hershey, PA USA

**Keywords:** Proton Pump inhibitor, Acute kidney Injury, Prospective matched cohort study, Post-hospitalization

## Abstract

**Background:**

Proton Pump Inhibitors (PPI) are among the most commonly used drugs to treat acid-related gastrointestinal disorders in the USA. Although PPI use has been linked to acute interstitial nephritis, the side effects of post-hospitalization acute kidney injury (AKI) and the progression of kidney disease still are controversial. We conducted a matched cohort study to examine the associations between PPI use and the side effects, especially in post-hospitalization AKI.

**Methods:**

We investigated 340 participants from the multicenter, prospective, matched-cohort ASSESS-AKI study, which enrolled participants from December 2009 to February 2015. After the baseline index hospitalization, follow-up visits were conducted every six months, and included a collection of self-reported PPI use by participants. Post-hospitalization AKI was defined as the percentage increase from the nadir to peak inpatient SCr value was ≥ 50% and/or absolute increase ≥ 0.3 mg/dL in peak inpatient serum creatinine compared with baseline outpatient serum creatinine. We applied a zero-inflated negative binomial regression model to test the relationship between PPI use and post-hospitalization AKI. Stratified Cox proportional hazards regression models also were conducted to examine the association between PPI use and the risk of progression of kidney disease.

**Results:**

After controlling for demographic variables, baseline co-morbidities and drug use histories, there was no statistically significant association between PPI use and risk of post-hospitalization AKI (risk ratio [RR], 0.91; 95% CI, 0.38 to 1.45). Stratified by AKI status at baseline, no significant relationships were confirmed between PPI use and the risk of recurrent AKI (RR, 0.85; 95% CI, 0.11 to 1.56) or incidence of AKI (RR, 1.01; 95% CI, 0.27 to 1.76). Similar non-significant results also were observed in the association between PPI use and the risk of progression of kidney diseases (Hazard Ratio [HR], 1.49; 95% CI, 0.51 to 4.36).

**Conclusion:**

PPI use after the index hospitalization was not a significant risk factor for post-hospitalization AKI and progression of kidney diseases, regardless of the AKI status of participants at baseline.

**Supplementary Information:**

The online version contains supplementary material available at 10.1186/s12882-023-03211-4.

## Introduction

Acute kidney injury (AKI) is a significant complication for hospitalized patients and is related to severe short-term and long-term mortality and morbidity, including increased risk of death, progression of kidney disease, and heart failure [1, 2]. Post-hospitalization AKI, including the new incidence of AKI and recurrent AKI, is a common sequela after one AKI episode and is associated with incident chronic kidney disease (CKD), progression of pre-existing CKD, and end-stage kidney disease (ESKD) [3]. Potential risk factors for new incidence and recurrent AKI include older age, African ancestry and chronic comorbidities, such as congestive heart failure (CHF), and diabetes [4, 5].

Proton pump inhibitors (PPI) are widely used for acid suppression therapy in the USA, with over 15 million adults using PPI every year [6]. PPIs are commonly prescribed to treat acid-related gastrointestinal disorders, including gastroesophageal reflux disease, esophageal strictures, Barrett’s esophagus, and peptic ulcers [7–9]. Kidney disease is one of the side effects of using PPI. Occurrence of acute interstitial nephritis (AIN) has been histologically confirmed as an adverse event of PPI use [7, 10–12]. AKI is observed as a potential adverse effect of PPI use, while the mechanism behind it still is obscure [10].

Previous epidemiological studies investigating the relationship between PPI use and AKI mainly were based on retrospective cohort analyses of data collected as part of routine clinical care [13]. These studies also are limited in their interpretation because they focused only on the incidence of AKI [13], only focused on death or kidney disease progression [14, 15], and rarely addressed recurrent AKI. In an effort to address these concerns, to the best of our knowledge, our study is the first to employ a matched prospective cohort design to investigate relationship between PPI use and multiple AKI counts during follow-up periods. We conducted this study with a large prospective cohort to test two hypotheses: (1) PPI use is related to the risk of post-hospitalization AKI, including incidence of AKI and recurrent AKI; (2) PPI use is associated with kidney disease progression.

## Methods

### Study population

The Assessment, Serial Evaluation, and Subsequent Sequelae in AKI (ASSESS-AKI) is a prospective cohort study of participants with and without an episode of AKI, who attended a baseline study visit 3 months after the index hospitalization [16, 17]. Participants were enrolled from four North American centers between December 2009 and February 2015. Follow-up in-person study visits were conducted 3 and 12 months after the index hospitalization and annually after that through November 2018, with a determination of estimated glomerular filtration rate (eGFR) and serum creatinine concentration (SCr) [18]. In addition, telephone visits were conducted six months after each annual visit. If participants were hospitalized, then medical records including all in-patient creatinine determinations were obtained. Medical history, study events, and use of medications were updated at each in-person visit or phone contact. Detailed study design and eligibility criteria were included in the supplement and have been published previously [16].

**Fig. 1:**
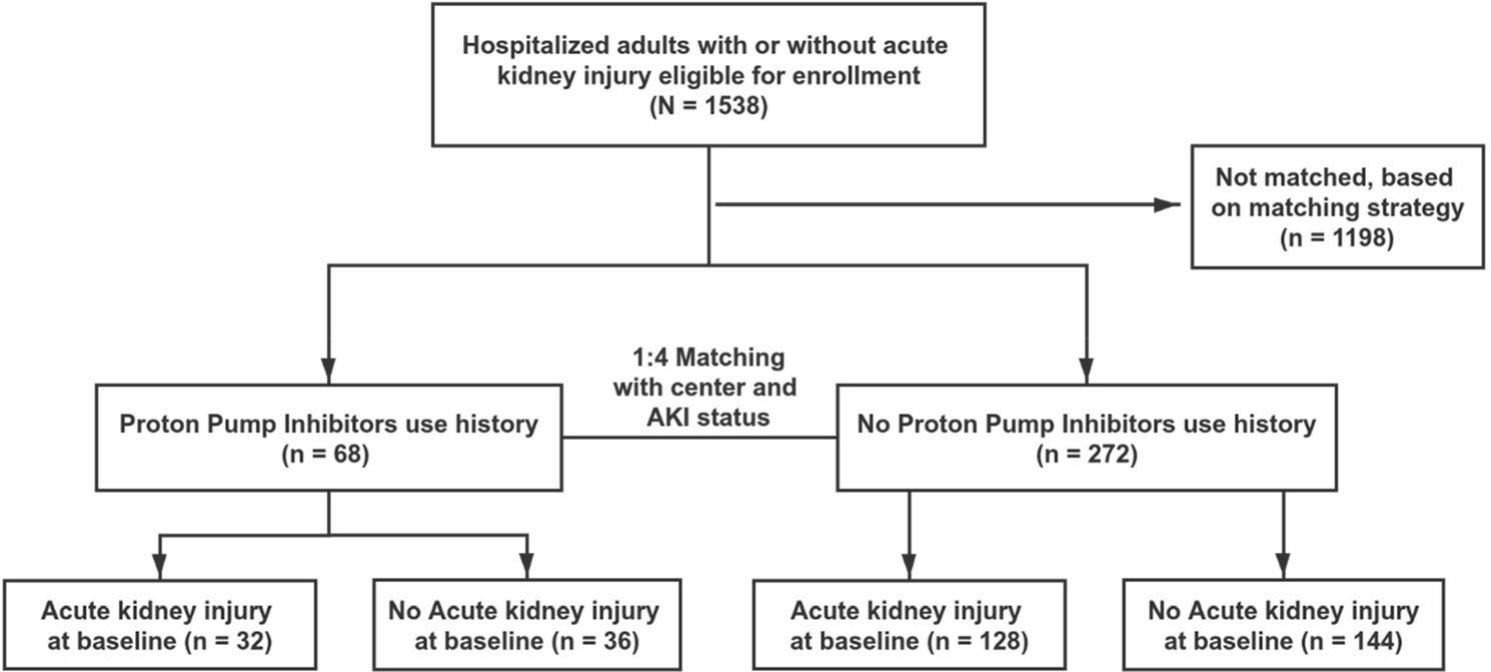
Assembly of matched cohort of adults surviving a hospitalization with and without PPI use

For this analysis, we matched participants with history of PPI use to participants without PPI use during the follow-up period (Fig. 1). The exact matching strategy was performed first with matching factors of center and baseline AKI status. Under each subgroup, we used propensity score matching strategy to deal with further potential confounders [19]. The propensity score analysis was conducted using a multivariable logistic regression to model PPI use during the follow-up period as a function of 13 covariates, including age, gender, race, intensive care unit (ICU) history, creatinine at baseline, diabetes mellitus history, cardiovascular disease history, hypertension history, and six drugs use at baseline (Angiotensin-converting enzyme (ACE) inhibitors, angiotensin receptor blockers (ARB), anti-hypertensive agents, diuretics, insulin, and statins). The nearest-neighbor matching was used, and 1:4 matching was performed with the “without replacement” sampling method. We regarded standardized mean difference (SMD) as a measure to evaluate the matching results [20].

Institutional review boards at the participating centers (Data Coordinating Center: Pennsylvania State University, and Clinical Research Centers: Yale University, Kaiser Permanente of Northern California, Vanderbilt University, University of Washington) approved the ASSESS-AKI study, and all methods were conducted in accordance with relevant guidelines and regulations.

### Assessment of proton pump inhibitor use

Participants were identified as PPI users if they met both of two criteria: (1) participant’s self-reported or medical-recorded use of PPI (Supplemental Table 1) at any follow-up study visits; (2) at least one visit with PPI use occurring earlier than the incidence of AKI.

### Ascertainment of outcomes

Post-hospitalization AKI was counted as one time if participants were in hospitalization with AKI records, which was defined as the percentage increase from the nadir to peak inpatient SCr value was ≥ 50% and/or absolute increase ≥ 0.3 mg/dL (26 µmol / L) in peak inpatient serum creatinine compared with baseline outpatient serum creatinine. For two consecutive episodes of AKI to be considered distinct episodes, they have to meet criteria for non-AKI (i.e., minimum of two serum creatinine in between and ≤ 0.2 mg/dL change above baseline) between episodes. Meanwhile, two consecutive episodes should be separated by at least 30 days [16].

We defined the incidence of AKI when participants without AKI at baseline were diagnosed with AKI during the follow-up period. Recurrent AKI was defined as participants with AKI at baseline who were diagnosed as AKI at least once during the follow-up period.

Progression of kidney disease was included in this study as a secondary outcome. Progression of kidney disease was defined as the occurrence of ESKD (a recipient of outpatient maintenance dialysis or a kidney transplant) or halving of eGFR since the time of the baseline study visit [16].

### Assessment of covariates

Demographic information and comorbidities were collected at the baseline study visit by participants’ self-report. All SCr results were performed using an isotope dilution mass spectrometry–traceable assay. eGFR was estimated using the Chronic Kidney Disease Epidemiology Collaboration (CKD-EPI) estimating equation [21]. Drug use information on a participant at baseline was collected during index hospitalization.

### Power justification

We performed a simulation study to assess the effect size that can be detected with 80% empirical power [24]. Utilizing binomial and negative binomial distributions, we simulated 1:4 matched datasets comprising 340 participants, mirroring our actual study. We subsequently applied the Zero-Inflated Negative Binomial (ZINB) model to analyze these simulated datasets. This simulation process was repeated 10,000 times, and we assessed the effect size (rate ratio) with statistically significant p-values (< 0.05) on approximately 8,000 occasions.

### Statistical analysis

For the 340 participants in this cohort study (68 PPI users and 272 PPI non-users), we summarized baseline participant characteristics across PPI users and non-users groups, with mean (SD) values for continuous variables, number and percentage for categorical variables.

For the primary analysis, after examining the hyper-Poisson variability of AKI counts (mean [SD] counts 0.71 [2.15]) and the dispersion statistic ((Pearson statistic)/(degree of freedom) = 1.66), an overdispersion of AKI counts was confirmed and negative binomial regression model would be an appropriate method [22]. Zero-inflation was confirmed in that 237 (69.7%) participants displayed zero counts of AKI incidence (Supplemental Table 2), and a significant positive Vuong statistic was presented (Z = 6.54, P < 0.0001). Therefore, we extended the negative binomial regression model to the ZINB regression model in order to account for the over-abundance of zeros [23].

After confirming no violations of the proportional hazards assumption, Cox proportional hazards regression models were conducted to examine the association between PPI use and the risk of progression of kidney disease, stratified by the matched covariates. For progression of kidney disease, participants were right-censored for death, loss to follow-up, or end of the study, whichever came first.

### Sensitivity analyses

We performed four distinct sensitivity analyses to evaluate the robustness of our findings under various scenarios.

Scenario 1: Sensitivity analyses were performed by including covariates into the regression models, conducting multivariable ZINB regression analysis for post-hospitalization AKI, and multivariable Cox-proportional hazards regression analysis for progression of kidney disease.

Scenario 2: Comorbidities were considered as time-dependent covariates, and we aimed to evaluate the impact of newly developed comorbidities during the follow-up period on the hazard ratios (HR). In this context, post-hospitalization AKI was analyzed as time-to-event data, and a multivariable stratified Cox proportional hazards regression model was employed to examine the HR associated with PPI use and AKI. Concurrently, diabetes, cardiovascular disease, and hypertension were incorporated into the regression model as time-dependent variables [25].

Scenario 3: We matched six drug use histories, although there were additional medications, such as non-steroidal anti-inflammatory drugs (NSAIDs), aspirin, vasopressors, immunosuppressants, corticosteroids, and chemotherapeutics, that might influence the relationship between PPI use and AKI. To ensure optimal matching outcomes, we excluded these medications during the matching process. However, we were able to incorporate these drug use histories into the multivariable ZINB regression model.

Scenario 4: For individuals who developed AKI 6 months after discontinuing PPI use, we excluded these PPI users (N = 3) and their four matched pairs (N = 12) from our regression analysis.

We used R software version 3.6.2 (R Foundation for Statistical Computing, Vienna, Austria) for the power simulation and matching process, and SAS software (Version 9.4, SAS Institute, Cary, NC) for further analyses with a two-tailed alpha level of 0.05.

## Results

### Participants characteristics

The cohort of 340 eligible participants included 68 who used PPI and 272 who did not use PPI during the follow-up periods, with a median follow-up of 4.9 years (IQR, 3.6–6.0 years; maximum follow-up, 7.8 years). Before matching, there were imbalanced distributions within the ASSESS-AKI data set (68 PPI users and 1470 PPI non-users) with respect to center (SMD = 0.316), gender (SMD = 0.109), race (SMD = 0.346), age (SMD = 0.273), hypertension (SMD = 0.159), diuretics use (SMD = 0.218) and insulin use (SMD = 0.147). After matching, the SMD of all these covariates became less than 0.1, indicating well-balanced distributions of each variable across 68 PPI users and 272 PPI non-users *(*Table 1*)*.

**Table 1:** Characteristics of Participants Cohorts Before and After Matching

Characteristic	Before matching			After 1:4 matching		
	**No PPI**	**PPI**	SMD*	**No PPI**	**PPI**	SMD*
	**(N = 1470)**	**(N = 68)**		**(N = 272)**	**(N = 68)**	
**Age, y**			0.273			0.034
Mean (SD)	64.4 (12.7)	68.0 (13.4)		68.4 (11.8)	68.0 (13.4)	
**Center**			0.316			< 0.001
Yale	286 (19.5%)	22 (32.4%)		88 (32.4%)	22 (32.4%)	
Vanderbilt	484 (32.9%)	18 (26.5%)		72 (26.5%)	18 (26.5%)	
Kaiser Permanente	298 (20.3%)	14 (20.6%)		56 (20.6%)	14 (20.6%)	
UW	402 (27.3%)	14 (20.6%)		56 (20.6%)	14 (20.6%)	
**Gender**			0.109			0.008
Female	552 (37.6%)	22 (32.4%)		94 (34.6%)	22 (32.4%)	
**Race**			0.346			< 0.001
White	1201 (81.7%)	59 (86.8%)		236 (86.8%)	59 (86.8%)	
Black/African American	186 (12.7%)	9 (13.2%)		36 (13.2%)	9 (13.2%)	
Other	83 (5.7%)	0 (0%)		0	0	
**Baseline AKI status**			0.062			< 0.001
Yes	737 (50.1%)	32 (47.1%)		128 (47.1%)	32 (47.1%)	
**ICU**			0.065			0.016
Yes	971 (66.1%)	47 (69.1%)		186 (68.4%)	47 (69.1%)	
**Diabetes**			0.065			0.008
Yes	631 (42.9%)	27 (39.7%)		109 (40.1%)	27 (39.7%)	
**CVD**			0.042			0.044
Yes	661 (45.0%)	32 (47.1%)		134 (49.3%)	32 (47.1%)	
**Hypertension**			0.159			0.08
Yes	1100 (74.8%)	46 (67.6%)		194 (71.3%)	46 (67.6%)	
**Creatinine**			0.059			0.002
Mean (SD)	1.79 (1.47)	1.72 (1.02)		1.71 (1.23)	1.72 (1.02)	
**Other Drug use at baseline**						
ACE Inhibitors use	492 (33.5%)	22 (32.4%)	0.024	91 (33.5%)	22 (32.4%)	0.023
ARB use	238 (16.2%)	9 (13.2%)	0.083	30 (11.0%)	9 (13.2%)	0.068
Antihypertensive agents use	1021 (69.5%)	47 (69.1%)	0.007	195 (71.7%)	47 (69.1%)	0.056
Diuretics use	653 (44.4%)	23 (33.8%)	0.218	94 (34.6%)	23 (33.8%)	0.016
Insulin use	298 (20.3%)	10 (14.7%)	0.147	42 (15.4%)	10 (14.7%)	0.021
Statins use	843 (57.3%)	38 (55.9%)	0.030	163 (59.9%)	38 (55.9%)	0.082

Abbreviation: SMD, standardized mean difference; ACE, Angiotensin-converting enzyme; ARB, Angiotensin receptor blockers

*An SMD greater than 0.1 is a threshold recommended for declaring imbalance

We assessed the median duration from cohort start, expressed in months, until the onset of post-hospitalization AKI for the study participants (Table 2). The overall median time to post-hospitalization AKI was 17.5 months. Among participants who experienced AKI during the baseline period, the median duration was 13.2 months. For those without baseline AKI, the median time to post-hospitalization AKI extended to 35.9 months. Furthermore, we evaluated the median duration, in months, until the progression of kidney disease for the study participants (Table 2). The overall median time to kidney disease progression was 39.1 months. For participants who experienced AKI during the baseline period, the median duration was 31.5 months, while those without baseline AKI demonstrated a longer median time of 46.0 months. Moreover, we evaluated the median duration of PPI use, which was found to be 45.3 months (interquartile range: 13.4 months at the first quartile and 62.9 months at the third quartile).

### Association between PPI use and the risk of post-hospitalization AKI

After controlling for demographic variables, baseline co-morbidities and drug use histories, there was no statistically significant association between PPI use and risk of post-hospitalization AKI (rate ratio [RR], 0.91; 95% CI, 0.38 to 1.45). Stratified by AKI status at baseline, no significant relationships were confirmed between PPI use and the risk of recurrent AKI (RR, 0.85; 95% CI, 0.11 to 1.56) or incidence of AKI (RR, 1.01; 95% CI, 0.27 to 1.76) *(*Table 2*).*

### Outcome of kidney disease progression

After controlling for demographic variables, baseline co-morbidities and drug use histories, there was no statistically significant association between PPI use and risk of kidney disease progression (Hazard Ratio [HR], 1.49; 95% CI, 0.51 to 4.36). Similar non-significant results also were observed in participants with AKI at baseline (HR 2.41, 95% CI, 0.68 to 8.61) and participants without AKI at baseline (HR 1.20, 95% CI 0.24 to 5.96) *(*Table 2*).*

**Table 2:** Association of PPI use at 3 months post-discharge and subsequent adverse outcomes

Events	Median months (Q1, Q3) *	PPI use N (%)	PPI non-use N (%)	RR or HR (95% CI) **
Post-hospitalization AKI				
All participants	17.5 (6.8, 41.6)	21 (30.9%)	82 (30.1%)	0.91 (0.38, 1.45)
AKI at baseline	13.2 (6.0, 31.2)	11 (34.4%)	49 (38.3%)	0.85 (0.11, 1.56)
No AKI at baseline	35.9 (13.3, 51.9)	10 (27.8%)	33 (22.9%)	1.01 (0.27, 1.76)
**Progression of kidney disease**				
All participants	39.1 (15.2, 53.5)	6 (8.8%)	13 (4.8%)	1.49 (0.51, 4.36)
AKI at baseline	31.5 (10.0, 38.7)	4 (12.5%)	7 (5.5%)	2.41 (0.68, 8.61)
No AKI at baseline	46.0 (41.6, 64.4)	2 (5.6%)	6 (4.2%)	1.20 (0.24, 5.96)

* Median months from cohort start to incidence of events, with 1st quartile (Q1) and 3rd quartile (Q3)

** For the events of post-hospitalization AKI, the measure is Rate Ratio (RR) with 95% confidence interval (CI)

For the events of progression of kidney disease, the measure is Hazard Ratio (HR) with 95% confidence interval (CI)

Abbreviations: PPI: Proton Pump Inhibitor; AKI: Acute Kidney Injury

### Power justification results

Throughout the 10,000 simulation iterations, approximately 8,000 of the simulated data sets resulted in statistically significant rate ratios (P < 0.05). At this point, we achieved 80% empirical power. With 68 participants in the exposure group and 272 participants in the matched control group, we determined the detectable effect size (rate ratio) to be 1.40 in our study.

### Sensitivity analyses

Sensitivity analyses were conducted across four scenarios, and the outcomes consistently aligned with our primary findings (Supplemental Table 3).

## Discussion

This large prospective cohort study showed that PPI use after discharge from the hospital was not related to post-hospitalization AKI (RR, 0.91; 95% CI, 0.38 to 1.45), including the risk of recurrent AKI (RR, 0.85; 95% CI, 0.11 to 1.56) and incidence of AKI (RR, 1.01; 95% CI, 0.27 to1.76). Our results are consistent with a recent self-controlled case series study. After controlling for drug use history and demographic information, PPI was not a risk factor for the incidence of AKI (adjusted rate ratio (aRR), 0.82; 95% CI, 0.60 to 1.13), with 3,685 participants [26].

However, some previous retrospective cohort studies have shown that PPI use leads to the incidence of AKI based on retrospective data analysis [13, 27–29]. These studies used large population datasets, such as 93,335 participants in the population-based health-maintenance organization (HMO) [13], 10,482 participants in the Atherosclerosis Risk in Communities (ARIC) study [27], 290,592 participants in a population-based cohort study in Canada [28], and claims data from a private health insurer with 184,480 participants in USA [29]. The incidence of AKI in these studies was measured as a binary outcome or time-to-event outcome with ICD-9 or ICD-10 codes, and the logistic regression model and Cox proportional hazards regression model were performed to estimate the adjusted odds ratio (aOR, 4.35; 95% CI, 3.14 to 6.04) [13], (aOR, 2.25; 95% CI 1.09 to 4.62) [29], and the adjusted hazard ratio (aHR, 1.31; 95% CI, 1.22 to 1.42) [27], (aHR, 2.52; 95% CI 2.27 to 2.79) [28].

In contrast to previous retrospective studies, we attribute the differences in our study’s results to several distinct perspectives, which also represent the novelty of our investigation. First, our study has strengths in study design, since we executed a systematically organized prospective cohort study with a structured research protocol, reducing ascertainment bias and minimizing the level of missing data. A valid estimation of AKI was conducted with longitudinal measurement of SCr to define the counts of recurrent AKI, instead of the classification code of disease. Additionally, we performed four sensitivity analyses to assess the robustness of our findings under various scenarios, including alternative statistical methods, the inclusion of three time-dependent covariates, the addition of six more medication use histories, and the exclusion of individuals with a long gap between PPI use and the onset of post-hospitalization AKI. The conclusions drawn from these four scenarios remained consistent with our primary analysis. To the best of our knowledge, our study is the first to examine the effect of time-dependent covariates on the relationship between PPI use and post-hospitalization AKI. The status of comorbidities can change over time; however, previous studies have only adjusted or matched for baseline covariates [13, 27–29]. Sensitivity analysis scenario 2 minimized this concern by incorporating time-dependent variables, including diabetes, cardiovascular disease, and hypertension, into the Cox proportional hazards regression model.

Second, we performed a mixed matching strategy to guarantee balanced distributions of covariates across the PPI use and non-use group. Besides demographic information and comorbidities, other drug use histories also are included as covariates of the propensity score matching strategy. Current studies found that ACE inhibitors [30], angiotensin receptor blockers [30], anti-hypertensive agents [31], diuretics [32, 33], insulin [34], and statins [35] are potential risk factors for AKI or other kidney diseases. However, previous populational retrospective studies of PPI and AKI rarely controlled for the effects of these drugs. In our research, we minimized the potential confounding effects of these medications through matching, and provided more accurate estimations of the relationship between PPI use and post-hospitalization AKI. One previous study showed the confounding effects of drug use history on the relationship between PPI use and the incidence of AKI. Before controlling for drug use history, AKI was associated with PPI use. However, after adjusting for macrolide antibiotics use, PPI was not a risk factor for the incidence of AKI [26].

Third, our study is the first one to analyze AKI as a count outcome to measure recurrent times, instead of a binary or time-to-event outcome as in previous studies. We believe we performed an appropriate estimation of the risk of recurrent AKI and our analysis is the first to use the ZINB regression model to measure the association between PPI use and the risk of recurrent AKI. We first tested the overdispersion of data to choose the negative binomial model instead of the Poisson model. Then, we expanded the negative binomial model to the ZINB model with “SAS PROC NLMIXED” [36] to estimate RR, after examining the zero-inflation effects of the data. We also compared the dispersion statistics [22], AIC, AICC, and BIC among the negative binomial model, zero-inflated Poisson (ZIP) model, and ZINB model. ZINB regression model was finally selected, and we believe the ZINB model should be considered for future recurrent AKI-related analyses.

In our study, there was no statistically significant association between PPI use and risk of kidney disease progression, while most previous studies showed PPI as a significant harmful factor for kidney disease progression [13, 37]. Participants in our study were provided access to more professional medication instructions and supervision than those in the population retrospective studies. Previous studies suggested that monitoring of kidney function was essential to lower the risk of adverse events of PPI [7]. The ASSESS-AKI study was a prospective cohort study in which participants underwent annual clinic visits to assess their health status and receive appropriate health guidance. The individuals included in retrospective data analyses do not always have that opportunity. Our analysis strongly suggests that judicious use of PPI with instructions from health care providers could decrease potential risks of unwanted side effects [7].

A clinical concern regarding our study is the possibility of insufficient PPI exposure, which could result in a non-significant impact on post-hospitalization AKI. Unlike retrospective studies, which can trace back to the initiation of PPI use, our prospective study can only capture the PPI use period after participant recruitment. We did not collect information as to when participants began using PPIs or their reasons for requiring PPIs. In our cohort, the median PPI exposure duration was 3.8 years (interquartile range, 1.1–5.2). This exposure period is comparable to a previous study that reported 3.9 years (interquartile range, 3–4.2) and demonstrated a significant HR of 2.89 (95% CI, 1.91–4.38) for PPI use in relation to AKI events [15]. Therefore, PPI users in our study have a similar exposure period to those in previous research. An additional concern pertains to the dosages of PPI use in our study. A previous investigation indicated that higher PPI dosages are associated with increased hazard ratios for kidney diseases compared to standard dosages [38]. Regrettably, we did not gather dosage information in our cohort, preventing us from assessing the effect of PPI dosage on post-hospitalization AKI outcomes.

Our study has several limitations. First, the relative small sample size in the PPI use group (N = 68) yields low statistical power [39]. This partially explains the relatively large confidence interval of estimations. Nevertheless, our power simulation study reveals that our study possesses adequate power (80%) to detect a rate ratio of 1.40. Previous studies have indicated that the OR and HR for PPI use exceed 2.0 [13], [28], [29]. Thus, if the OR or HR values from previous studies hold true, our study is also capable of detecting a significant association. Second, although we matched participants with demographic, comorbid and drug use histories, the residual confounding effects still are possible. Third, in our study, we evaluated the incidence of AKI during the follow-up period by analyzing hospitalization records. It should be noted that our analysis may not account for cases managed exclusively in outpatient settings, potentially leading to an underestimation of the overall AKI prevalence. Moreover, our study did not distinguish between continuous PPI use after the index of hospitalization and new prescriptions of PPI. Although we examined the pattern of PPI use in the follow-up cohorts, we did not know the reasons for PPI initiation, continuation, or discontinuation. Thus, we may miss some interactions or confounders with PPI use. Finally, as explained above, we did not measure the dose and length of PPI use. Therefore, our analyses did not involve the relationship between cumulative exposures to PPI and outcomes.

## Conclusion

With a prospective cohort study of 340 participants, after adjusting for demographic variables, baseline co-morbidities and drug use histories, PPI use after the index hospitalization was not a significant risk factor for post-hospitalization AKI and progression of kidney diseases, regardless of the AKI status of participants at baseline.

## Electronic supplementary material

Below is the link to the electronic supplementary material.

Supplementary Material 1

## Data Availability

The data analyzed in the current study are available in the NIDDK Central Repository: https://repository.niddk.nih.gov/studies/assess-aki/.
