# Supplementary material for: The association between proton pump inhibitor use and risk of post-hospitalization acute kidney injury: a multicenter prospective matched cohort study
Source: BMC Nephrol. 2023 May 26;24:150. doi: 10.1186/s12882-023-03211-4 (PMC10224298; doi:10.1186/s12882-023-03211-4)
Supplement: Supplementary file 1 — Supplementary Material 1 [file 12882_2023_3211_MOESM1_ESM.docx]

| Supplemental Table 1. ASSESS-AKI Study PPI list during follow-up periods |
| --- |
| Generic Name |
| Esomeprazole Magnesium |
| Esomeprazole Sodium |
| Lansoprazole |
| lansoprazole (Iv) |
| lansoprazole; naproxen |
| Rabeprazole Sodium |
| Pantoprazole Sodium |
| Pantoprazole Sodium (Iv) |
| Dexlansoprazole |
| Magnesium Hydroxide; Omeprazole; Sodium Bicarbonate |

| Supplemental Table 2. Post-hospitalization AKI counts distribution across PPI status after 3-month post-discharge | | | | | | |
| --- | --- | --- | --- | --- | --- | --- |
| PPI use | 0 | 1 | 2 | 3 | 4 | Total |
| Yes | 47 | 10 | 8 | 3 | 0 | 68 |
|  | 69.12% | 14.71% | 11.76% | 4.41% | 0.00% |  |
| No | 190 | 57 | 17 | 5 | 3 | 272 |
|  | 69.85% | 20.96% | 6.25% | 1.84% | 1.10% |  |
| Total | 237 | 67 | 25 | 8 | 3 | 340 |

| Supplemental Table 3. Sensitivity analyses for the relationship between PPI use and post-hospitalization AKI under four scenarios | | | |
| --- | --- | --- | --- |
| Post-hospitalization AKI (RR or HR, 95% CI) | All Participants | AKI at baseline | No AKI at baseline |
| Scenario 1*: Multivariable regression with covariates same as matching | 0.81 (0.51, 1.27) | 1.02 (0.50, 2.08) | 0.75 (0.41, 1.38) |
| Scenario 2**: Multivariable regression with time-dependent comorbidities | 1.01 (0.52, 1.96) | 1.08 (0.67, 1.76) | 1.18 (0.58, 2.40) |
| Scenario 3***: Multivariable regression with further drug use histories | 1.17 (0.71, 1.91) | 1.01 (0.52, 1.98) | 1.33 (0.64, 2.73) |
| Scenario 4****: Stratified regression excludes participants with extended gaps between discontinuing PPI use and the incidence of AKI | 0.88 (0.51, 1.51) | 0.86 (0.42, 1.77) | 1.06 (0.51, 2.22) |
|  |  |  |  |
| * ZINB model adjusted for age, gender, race, intensive care unit (ICU) history, creatinine at baseline, diabetes mellitus history, cardiovascular disease history, hypertension history, and six drugs used at baseline (Angiotensin-converting enzyme inhibitors, angiotensin receptor blockers, anti-hypertensive agents, diuretics, insulin, and statins).  ** Cox Proportional hazard regression models adjusted for time-dependent diabetes, time-dependent cardiovascular disease, and time-dependent hypertension, stratified with match ID.  ***ZINB model adjusted for non-steroidal anti-inflammatory drugs (NSAIDs), aspirin, vasopressors, immunosuppressants, corticosteroids, and chemotherapeutics use histories, and stratified with match ID.  ****ZINB model excludes PPI users who developed AKI 6 months after discontinuing PPI use, and their four matched pairs. | | | |

Abbreviations: PPI: Proton Pump Inhibitor; AKI: Acute Kidney Injury; ZINB: Zero-inflated negative binomial model;

RR: Rate ratio; HR: Hazard Ratio
